# Psilocybin does not induce the vulnerability marker HSP70 in neurons susceptible to Olney’s lesions

**DOI:** 10.1007/s00406-023-01699-3

**Published:** 2023-11-07

**Authors:** Ana-Maria Iorgu, Andrei-Nicolae Vasilescu, Natascha Pfeiffer, Rainer Spanagel, Anne Stephanie Mallien, Dragos Inta, Peter Gass

**Affiliations:** 1 Department of Psychiatry and Psychotherapy, Research Group Animal Models in Psychiatry, Central Institute of Mental Health, Medical Faculty Mannheim, University of Heidelberg, J5, 68159 Mannheim, Germany; 2 Institute of Psychopharmacology, Central Institute of Mental Health, Medical Faculty Mannheim, University of Heidelberg, Mannheim, Germany; 3 Department for Community Health, Faculty of Natural Sciences and Medicine, University of Fribourg, Fribourg, Switzerland; 4 Department of Biomedicine, University of Basel, Basel, Switzerland

**Keywords:** Psilocybin, Ketamine, Antidepressant, HSP70, Olney’s lesions

## Abstract

S-ketamine, a N-methyl-D-aspartate receptor (NMDAR) antagonist, and psilocybin, a 5-hydroxy-tryptamine (serotonin) 2A receptor (5-HT_2A_R) agonist, are reported as effective rapid-acting antidepressants. Both compounds increase glutamate signalling and evoke cortical hyperexcitation. S-ketamine induces neurotoxicity especially in the retrosplenial cortex (Olney’s lesions). Whether psilocybin produces similar neurotoxic effects has so far not been investigated. We performed an immunohistochemical whole-brain mapping for heat shock protein 70 (HSP70) in rats treated with psilocybin, S-ketamine, and MK-801. In contrast to S-ketamine- and MK-801-treated animals, we did not detect any HSP70-positive neurons in retrosplenial cortex of rats treated with psilocybin. Our results suggest that psilocybin might be safer for clinical use compared to S-ketamine regarding neuronal damage.

## Introduction

Hallucinogens such as psilocybin and ketamine are reported as promising rapid-acting antidepressants [1, 2]. Ketamine, a non-competitive N-methyl-D-aspartate receptor (NMDAR) antagonist, showed antidepressant effects in numerous pre-clinical and clinical studies [1–4]. S-ketamine was recently approved by the FDA for clinical use in treatment-resistant depression [3, 4]. Despite its clinical utility, detrimental effects such as addiction, psychotic symptoms like hallucination, delusions, depersonalization, and cognitive deficits have been reported after repetitive ketamine treatment [1, 4].

Ketamine increases glutamate concentration in the brain [4–6]. This effect was proposed as a possible mechanism involved in the antidepressant activity of ketamine [1]. However, some authors claim that increased glutamate concentrations additionally enhance the risk of excitotoxicity and oxidative stress [3, 6]. Studies reported that ketamine might increase the levels of reactive oxygen species as well as might enhance inflammation, apoptosis, and autophagy [3]. High doses of ketamine and other NMDAR antagonists are neurotoxic and induce injury and neuronal cell death in retrosplenial cortex and posterior cingulate cortex [3, 7], leading to a characteristic neuropathologic lesion known as Olney’s lesion [3, 8, 9].

Preclinical [10] and clinical studies [11] indicate that psilocybin, a 5-hydroxy-tryptamine (serotonin) 2A receptor (5-HT_2A_R) agonist, might also be effective as a rapid-acting drug in treatment-resistant depression [1, 2]. Psilocin, a metabolite of psilocybin [12] and ketamine [13] were recently shown to promote brain-derived neurotrophic factor (BDNF) signaling via binding to BDNF receptor TrkB (neurotrophic receptor tyrosine kinase, Ntrk2) and by this mechanism to induce neuroplasticity. Recent studies in animal models [6] and neuroimaging studies in humans [14] revealed that psilocybin increases the extracellular level of glutamate in the brain, similar to ketamine [5, 6]*.* One study claimed that the antidepressant effect of psilocybin is independent of the 5-HT_2A_R agonism and that glutamatergic effects like changes in the α-amino-3-hydroxy-5-methyl-4-isoxazolepropionic acid receptor (AMPAR)/NMDAR and excitation/inhibition ratios may underlie its therapeutic efficacy [15]. Considering this, psilocybin might disturb the proportion between excitation and inhibition in cortical networks and lead to hyperexcitation in some cortical areas. Interestingly, a similar mechanism involving a disruption of the local excitatory/inhibitory networks with subsequent hyperexcitation was proposed for neurotoxicity of NMDAR antagonists in retrosplenial cortex [16] i.e., for Olney’s lesions, associated with induction of heat shock protein 70 (HSP70) [7]. Further studies showed that psilocybin elevates the oxidative DNA damage in frontal cortex and hippocampus [6], suggesting a potential risk by clinical use of psilocybin.

However, whether psilocybin indeed produces neurotoxic effects, has so far not been investigated experimentally. This is an important question, considering the potential large-scale clinical use of psilocybin in the future. Moreover, even though some authors claim that psilocybin might not be addictive and might be safe at therapeutic doses [11], adverse reaction such as prolonged psychotic symptoms, flashback phenomena, and hallucinogen-persisting perception disorder have also been reported [17]. The exact neurobiological mechanisms of these symptoms are still not elucidated. One possible hypothesis might be psilocybin-induced neurotoxicity.

To investigate whether psilocybin induces neurotoxic effects, we carried out a whole-brain mapping using immunohistochemical staining for HSP70, a well-known marker for stress reaction and cellular injury [18], in female Sprague–Dawley rats 24 h after treatment with psilocybin, S-ketamine, and the NMDAR antagonist MK-801. The region-specific expression of neuronal HSP70 in rat brain was analyzed via qualitative and quantitative analysis under light microscopy.

## Materials and methods

Experiments were performed in 12-week-old female Sprague–Dawley rats (Charles River) as in previous similar studies [7, 8, 19], since female rats were shown to be more sensitive to neurotoxicity induced by NMDAR antagonists [20]. Animals were treated intraperitoneally with: (1) vehicle (0.9% NaCl, 10 ml/kg), (2) S-ketamine 100 mg/kg (K1884, Sigma–Aldrich, Germany), (3) psilocybin 5 mg/kg, (4) psilocybin 20 mg/kg, and (5) MK-801 (dizocilpine) 1 mg/kg (ab120027, Abcam, Great Britain). Psilocybin was purchased by RS from Dr. Martin Kuchar, Forensic Laboratory of Biologically Active Compounds, Department of Chemistry of Natural Compounds, University of Chemistry and Technology Prague, Prague, Czech Republic. The validity of psilocybin produced by this laboratory was demonstrated in clinical studies showing acute psychedelic and long-term clinical effects and effects in PET neuroimaging in healthy individuals [21–28], as well as in animal studies [29, 30]. Doses of psilocybin (5 mg/kg and 20 mg/kg) were chosen according to Jefsen et al. (2021) [31]. The induction of HSP70 and immunoreactivity was reported in previous studies using NMDAR antagonists to be maximal 24 h after treatment [18].

24 h after treatment, rats were anesthetized with an overdose of ketamine (Serumwerk, Germany) and xylazin (WDT, Germany) and immediately after reaching the complete anesthetic state transcardially perfused with 4% paraformaldehyde (in 0.1 M phosphate buffer, pH 7.4). To avoid possible confounding effects of ketamine, transcardial perfusion and complete blood wash was performed in less than 5 min after the injection of ketamine. The brains were removed and postfixed in the same fixative for 24 h before vibratome sectioning into 40 μm thick coronal sections, as previously described [32]. All experiments were approved by German animal welfare authorities (Regierungspräsidium Karlsruhe) and complied with the European Communities Council Directive 2010/63/EU.

Coronal sections were incubated free-floating with an anti-HSP70 monoclonal antibody (Enzo Life Science, Germany, diluted 1:1000), as described earlier [32]. Immunoreactivity was visualized using avidin–biotin method and staining was developed using nickel-3,3-diaminobenzidine (DAB), as previously described [33]. Qualitative analysis of the HSP-70 expression was performed in every third section along the retrocaudal axis (at least 32 whole-brain slides per animal) using a light microscope (Leica TCS-NT). The complete surface of the whole-brain slide was examined. The brain regions were identified according to the atlas of the rat brain [34]. Quantification of HSP70-positive cells in the retrosplenial cortex was performed bilaterally at 40 × magnification in at least 10 whole-brain slides per animal. All HSP70-positive neuronal cells on the whole-brain slide situated in the region of interest (the retrosplenial cortex) were counted. The average number of HSP70-positive cells across all slides for each animal was determined. Data were visualized in RStudio using base R functions.

## Results

**Fig. 1:**
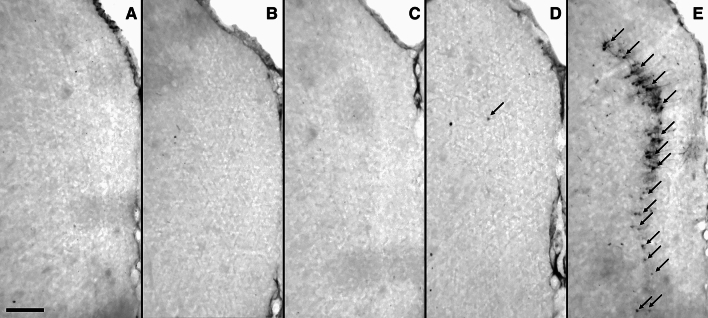
Representative cropped microscopic images showing the expression of HSP70 in retrosplenial cortex after treatment with **a** saline, **b** psilocybin 5 mg/kg, **c** psilocybin 20 mg/kg, **d** S-ketamine 100 mg/kg, and **e** MK-801 1 mg/kg. Note the prominent induction of HSP70 after MK-801-treatment and the weak induction in the S-ketamine-treated group, whereas no HSP70 expression is detected in the groups treated with psilocybin and saline. The arrows indicate HSP70-positive cells. Scale bar = 200 µm

**Fig. 2:**
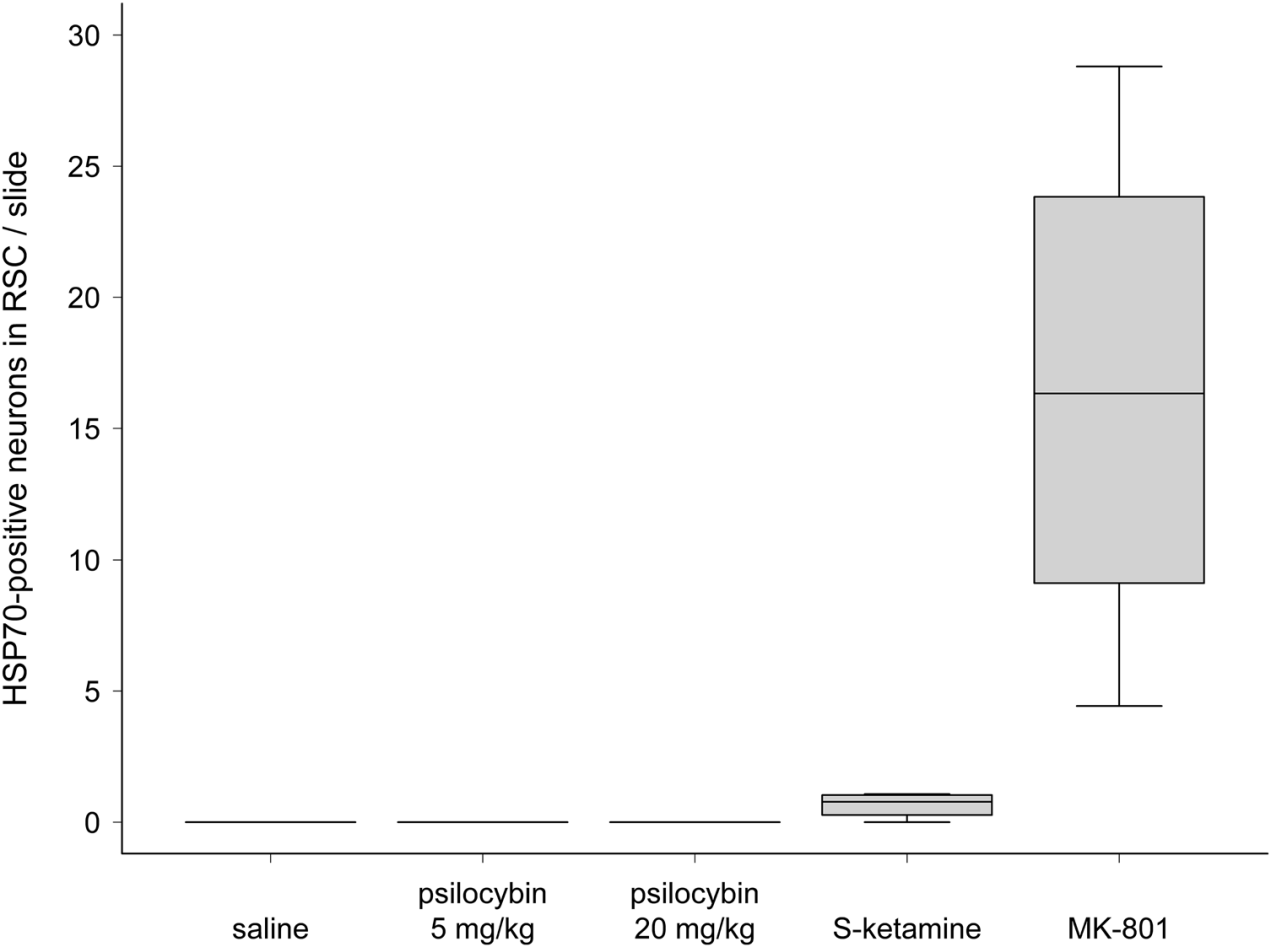
Quantitative analysis of HSP70-expression in the retrosplenial cortex. The boxplot shows the number of HSP70-positive cells in the retrosplenial cortex (RSC) per slide (median, 1st quartile, 3rd quartile, minimum, and maximum). HSP70-positive cells in the RSC were counted bilaterally on whole-brain slides in at least 10 slides per animal in the following groups: **a** saline (*n* = 4), **b** psilocybin 5 mg/kg (*n* = 6), **c** psilocybin 20 mg/kg (*n* = 6), **d** S-ketamine 100 mg/kg (*n* = 4), and **e** MK-801 1 mg/kg (*n* = 4)

In whole-brain mapping using immunohistochemistry, we did not detect any expression of HSP70 in the retrosplenial cortex in both groups treated with psilocybin (5 mg/kg and 20 mg/kg) 24 h after treatment (Fig. 1b and c), similar to the saline-treated, negative control group (Fig. 1a). In contrast, the S-ketamine-treated group revealed an induction of neuronal HSP70 in the retrosplenial cortex (Fig. 1d), which is in accordance with previous studies [7]. In the positive control group treated with the NMDAR antagonist MK-801, we observe an extensive expression of HSP70 in neurons in the retrosplenial cortex (Fig. 1e), as previously described [18]. Figure 2 illustrates the quantification of the HSP70-positive cells in the retrosplenial cortex in the experimental groups.

**Fig. 3:**
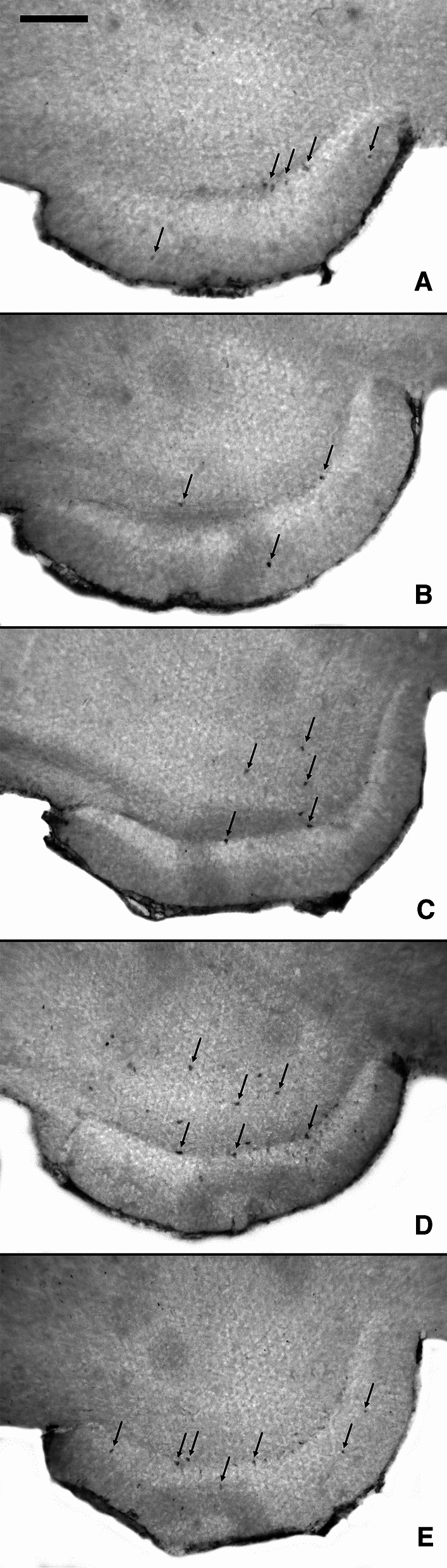
Representative cropped microscopic images showing the expression of HSP70 in hippocampus after treatment with saline **a** saline, **b** psilocybin 5 mg/kg, **c** psilocybin 20 mg/kg, **d** S-ketamine 100 mg/kg, and **e** MK-801 1 mg/kg. The arrows indicate HSP70-positive cells. Scale bar = 200 µm

Qualitative analysis of the HSP70-expression in the hippocampal region revealed in all experimental groups single or very few scattered immunostained neurons, without a visible difference between the groups. There was a weak expression of HSP70 in hippocampus in 2/4 animals in the negative control group (Fig. 3a), in 4/6 animals in the group treated with 5 mg/kg psilocybin (Fig. 3b), in 3/6 animals in the group treated with 20 mg/kg psilocybin (Fig. 3c), in 3/4 animals in the group treated with S-ketamine (Fig. 3d), and in 2/4 animals in the group treated with MK-801 (Fig. 3e). A quantitative assessment with statistical analysis did not seem constructive due to the very low number of weak immunopositive neurons detected in hippocampus—only single or very few positive cells—in only some animals of each experimental group. Our observation is consistent with previous histological studies in Sprague–Dawley rats, which detected a weak basal expression of HSP70 in hippocampal neurons even under unstressed conditions [35]. With exception of scattered weakly HSP70-expressing neurons in the hippocampus and the septal region similar in all groups, there was no further neuronal HSP70-expression in psilocybin-treated rats.

## Discussion

In this study, we demonstrate using whole-brain mapping for HSP70 that psilocybin does not increase the neuronal expression of HSP70 in any brain region, in contrast to S-ketamine and MK-801, indicating that psilocybin might not induce neurotoxicity.

HSP70 neuronal expression is widely recognized as a reliable marker of vulnerability after neuronal injury [18]. The neurotoxic effects of NMDAR antagonists like MK-801 and ketamine were analyzed in previous studies using the expression of HSP70 in neurons [7, 18]. In our present study, animals treated with MK-801 and S-ketamine revealed a distinct neuronal expression of HSP70 in retrosplenial cortex, as previously described [7, 18], indicating a stress response and neuronal injury in this brain region [18]. In contrast, psilocybin in both doses used did not induce an expression of HSP70 in neurons 24 h after treatment. Our results suggest that psilocybin in contrast to S-ketamine might not induce neurotoxicity and, consequently, that psilocybin might be a safer compound for clinical use compared to ketamine.

Both ketamine, a NMDAR antagonist, and psilocybin, a 5-HT_2A_R agonist, are reported to have similar rapid antidepressant effects, despite initial pharmacologic action on distinct receptors. Recent studies demonstrated that both psilocin, the active metabolite of psilocybin, and ketamine directly activate TrkB, the receptor of BDNF [12, 13]. Some authors proposed as shared mechanism of action an increased glutamatergic signalling with elevated concentrations of extracellular glutamate that activate AMPA receptors, which induce BDNF release and activation of mammalian target of rapamycin (mTOR) signalling, promoting neuroplasticity [1]. This common mechanism of action suggests that ketamine and psilocybin, and more generally NMDAR antagonists and 5-HT_2A_R agonists, might also exhibit similar detrimental effects. Supporting this, neurotoxicity was previously demonstrated for ketamine [7] and recently also reported for other 5-HT_2A_R agonists like the novel compound 25I-NBOMe [36]. However, using histological methods we did not observe in the present study neurotoxic effects in psilocybin-treated rats.

We chose the doses of psilocybin according to Jefsen et al. (2021), who used doses up to 20 mg/kg in rats without reporting any adverse effects or mortality [31]. Another study reported that a single dose of psilocybin 1 mg/kg induces long-lasting antidepressant effects in rats [10]. The doses of psilocybin used in our study (5 mg/kg and 20 mg/kg) are much higher than those having an antidepressant effect in animal models [10] and those used in clinical studies in humans, where the weight-adjusted dose of psilocybin ranges from 0.025 mg/kg to 0.42 mg/kg [11, 14, 21–23, 25]. Clinical studies reported that psilocybin in doses of around 0.2–0.3 mg/kg *per os* induces in healthy individuals acute subjective psychedelic effects such as altered state of consciousness, dissolution of ego and personhood, mystical experience, disembodiment, changed perception of time and space, audio-visual synesthesiae, positive mood, blissful state, reduction of sense of self and of the border between self and the external world, as well as long-term effects like increase in openness to new experiences and mindfulness, positive changes in mood, behavior and personality, increased cognitive flexibility [21, 23, 25]. A dose of 1 mg/kg psilocybin in Sprague–Dawley rats is equivalent to 0.16 mg/kg in humans [37]. We used in our study higher doses of psilocybin to detect even minimal neurotoxic effects. Even at the highest dose used (20 mg/kg) we did not detect any induction of HSP70 in neurons of retrosplenial cortex 24 h after treatment. We used this timepoint in our experiments since in previous studies using NMDAR antagonists the induction of HSP70 was reported to be maximal 24 h after treatment [18]. However, psilocybin changes could occur at later time points than 24 h after treatment, as investigated in this study. Further studies are, therefore, needed to investigate the potential long-term detrimental effects of psilocybin on neurons.

## Data Availability

The data that support the findings of this study are available from the corresponding author upon request.
